# Acidification increases abundances of *Vibrionales* and *Planctomycetia* associated to a seaweed-grazer system: potential consequences for disease and prey digestion efficiency

**DOI:** 10.7717/peerj.4377

**Published:** 2018-03-30

**Authors:** Tania Aires, Alexandra Serebryakova, Frédérique Viard, Ester A. Serrão, Aschwin H. Engelen

**Affiliations:** 1 Center for Marine Sciences (CCMAR), CIMAR, University of Algarve, Campus de Gambelas, Faro, Portugal; 2 Sorbonne Université, CNRS, Lab Adaptation and Diversity in Marine Environments (UMR 7144 CNRS SU), Station Biologique de Roscoff, Roscoff, France; 3 CNRS, UMR 7144, Divco Team, Station Biologique de Roscoff, Roscoff, France

**Keywords:** Invasive seaweeds, Ocean acidification, Grazer microbiomes, Algae microbiomes, Metabarcoding, *Sargassum muticum*, *Synisoma nadejda*

## Abstract

Ocean acidification significantly affects marine organisms in several ways, with complex interactions. Seaweeds might benefit from rising CO_2_ through increased photosynthesis and carbon acquisition, with subsequent higher growth rates. However, changes in seaweed chemistry due to increased CO_2_ may change the nutritional quality of tissue for grazers. In addition, organisms live in close association with a diverse microbiota, which can also be influenced by environmental changes, with feedback effects. As gut microbiomes are often linked to diet, changes in seaweed characteristics and associated microbiome can affect the gut microbiome of the grazer, with possible fitness consequences. In this study, we experimentally investigated the effects of acidification on the microbiome of the invasive brown seaweed *Sargassum muticum* and a native isopod consumer *Synisoma nadejda*. Both were exposed to ambient CO_2_ conditions (380 ppm, pH 8.16) and an acidification treatment (1,000 ppm, pH 7.86) for three weeks. Microbiome diversity and composition were determined using high-throughput sequencing of the variable regions V5-7 of 16S rRNA. We anticipated that as a result of acidification, the seaweed-associated bacterial community would change, leading to further changes in the gut microbiome of grazers. However, no significant effects of elevated CO_2_ on the overall bacterial community structure and composition were revealed in the seaweed. In contrast, significant changes were observed in the bacterial community of the grazer gut. Although the bacterial community of *S. muticum* as whole did not change, *Oceanospirillales* and *Vibrionales* (mainly *Pseudoalteromonas*) significantly increased their abundance in acidified conditions. The former, which uses organic matter compounds as its main source, may have opportunistically taken advantage of the possible increase of the C/N ratio in the seaweed under acidified conditions. *Pseudoalteromonas,* commonly associated to diseased seaweeds, suggesting that acidification may facilitate opportunistic/pathogenic bacteria. In the gut of *S. nadejda,* the bacterial genus *Planctomycetia* increased abundance under elevated CO_2_. This shift might be associated to changes in food (*S. muticum*) quality under acidification. *Planctomycetia* are slow-acting decomposers of algal polymers that could be providing the isopod with an elevated algal digestion and availability of inorganic compounds to compensate the shifted C/N ratio under acidification in their food.

In conclusion, our results indicate that even after only three weeks of acidified conditions, bacterial communities associated to ungrazed seaweed and to an isopod grazer show specific, differential shifts in associated bacterial community. These have potential consequences for seaweed health (as shown in corals) and isopod food digestion. The observed changes in the gut microbiome of the grazer seem to reflect changes in the seaweed chemistry rather than its microbial composition.

## Background

Ocean acidification significantly affects marine organisms in diverse ways (Fabry et al., 2008; Kroeker et al., 2013). In the case of species interactions (e.g., predator–prey), the outcome of such effects can be difficult to predict as antagonistic or synergistic effects may be observed (Asnaghi et al., 2013; Branch et al., 2013; Poore et al., 2013). This is particularly true for non-calcifying seaweeds, which in contrast to most other organisms can benefit from rising CO_2_ through increased photosynthesis and carbon acquisition, and subsequently acquire higher growth rates (Porzio, Buia & Hall-Spencer, 2011; Harley et al., 2012; Koch et al., 2013; Olischläger et al., 2013). However, changes in plant leaf chemistry in response to elevated carbon supply are expected to result in higher C:N and C:P ratios and, as such, reduce the nutritional quality of tissue for grazers (Urabe, Togari & Elser, 2003; Van De Waal et al., 2010) and the same is expected for seaweeds. Variations in the palatability of seaweeds may lead to changes in consumption rates by herbivores, which will have to absorb nutrients more efficiently or consume more to compensate for low concentrations of essential nutrients (Gutow et al., 2014). Thus, ocean acidification (OA) could have positive effects on seaweed growth rate, but may also induce behavioral changes on the herbivores and increased grazing rates. Therefore, it is important to understand the effects of ocean acidification on prey (bottom-up effects) and, as a top-down effect, on predation.

Interactions among organisms and their associated bacterial communities affect the holobiont physiology and health (Hollants et al., 2013; Egan et al., 2013), and play an important role in the functioning of hosts as, in the case of this study, seaweeds (Singh et al., 2011; Singh & Reddy, 2014). Seaweeds and marine organisms feeding on them live in a close association with diverse and abundant microbial communities (King et al., 2012; Hollants et al., 2013; Egan et al., 2013; Dudek et al., 2014). Seaweeds comprise dynamic species-specific bacterial communities (Aires et al., 2015; Aires, Serrão & Engelen, 2016; Vieira et al., 2016). The communities are recognized to have growth-promoting and nutritional effects (Head & Carpenter, 1975; Dimitrieva, Crawford & Yüksel, 2006), and to be involved in the production of biologically active (Chojnacka et al., 2012) and defensive (Burgess et al., 1999) compounds. At a higher trophic level, symbiotic bacteria inhabiting the guts of marine herbivores are also known to support important physiological functions (Hacquard et al., 2015), including the mediation of the digestion of food components by producing critical digestive enzymes for breaking down complex molecular structures (Mackie et al., 2004). In addition to digestive functions, grazers depend on seaweed-associated microbiota for nutrients found in the algal biofilm (i.e., proteins, polysaccharides, lipids, etc.; Tietjen, 2014). As such, diet represents an important factor in shaping microbial diversity in the intestinal systems of grazers. So, any changes in bacterial composition of the seaweed may result in diet-induced changes in the gut microbiota of grazers that may eventually affect their metabolism, as well as its fitness and biology (Mattila et al., 2014; Tietjen, 2014). Because carbon acquisition is expected to be facilitated for seaweeds at elevated CO_2_ levels, higher nutrient uptake is anticipated to help obtain other nutrients in the right balance with carbon. Part of these nutrients might be obtained through the microbiome and, therefore, the specific bacteria responsible for such acquisitions (e.g., phosphorous, nitrogen and iron) (Thomas et al., 2008; Burke et al., 2011b) might be positively selected and increase their abundance.

Because OA is expected to affect the interactions between marine herbivores and seaweeds through increased consumption of carbon enriched algal tissue (Gutow et al., 2014), the microbiomes of grazers might help with nutrient acquisition. While better understanding of the diversity and functions of associated symbiotic bacteria is needed, few studies have addressed the diversity and composition of gut microbiota of marine grazers (but see Hong et al., 2011; Devine, Pelletreau & Rumpho, 2012; Davis et al., 2013; Dudek et al., 2014).

To predict the responses of aquatic organisms to OA, it is necessary to understand responses of the host-associated microbiota to increasing CO_2_ and reduced pH. Little is known about the responses of associated microbiota to changes in pCO_2_ (partial pressure of carbon dioxide) including microbial metabolic capabilities or the ability to rapidly shift the host range (Morrow et al., 2015). Also, there is no consensus regarding whether a decrease in pH causes increase (Kerfahi et al., 2014), decrease (Taylor et al., 2014) or no changes (Hassenrück et al., 2016) in microbial richness and prevalence of dominant microbial taxa under acidification conditions. Furthermore, the current knowledge of acidification effects on the host-associated microbial communities is mostly based on the results of experiments conducted on corals. These experiments demonstrated that reduced pH initiates shifts in the coral microbiota towards microorganisms associated with stress and disease (Thurber et al., 2009; Meron et al., 2011; Webster et al., 2013). Therefore, there is a need for relevant studies on seaweeds, with a particular interest in species interactions as species may not respond similarly to OA and as effects can act synergistically. This response is particularly relevant to be examined in marine introduced seaweeds, because they are expected to benefit from future OA conditions, and thus increase their invasiveness.

In this study, we experimentally investigated the effects of acidification on the microbiomes of an emblematic invasive seaweed, the brown alga *Sargassum muticum*, and the gut microbiome of a native isopod consumer, *Synisoma nadejda*. This was done by following a three-week mesocosms exposure to elevated pCO_2_ followed by 16S amplicon sequencing in order to compare the bacterial community (hereafter microbiome) responses in these two hosts. Based on bacterial community characterization, our main hypotheses are that in acidified conditions (1) the seaweed-associated microbiome will have a different composition, (2) the grazers’ gut microbiome will mirror the changes in food source (assuming that seaweed nutritional content will change with acidification), when compared with ambient conditions. Considering the existing evidence for corals responses to OA (Thurber et al., 2009; Meron et al., 2011; Webster et al., 2013) and seaweeds responses to other environmental stresses (e.g., temperature, Case et al., 2011; Mensch et al., 2016) we expect seaweed microbiome to shift towards a community composed by stress related bacteria and putative pathogens. Also, raw plant consumers’ (e.g., fish, humans, etc.) gut microbiome and health is directly affected by environmental conditions (Sullam et al., 2012) or, following the “you are what you eat” premise, indirectly through changes in their food source/quality (Meziti et al., 2010; Berg et al., 2014), we predict that host’s “normal” gut bacterial composition could be affected by OA. An increased abundance of bacteria potentially assisting digestion could provide the isopod with an elevated algal digestion and availability of inorganic compounds to compensate the shifted C/N ratio under acidification in their food. Furthermore, if *S. muticum* showed a shift under OA conditions, we will investigate the presence of possible bacterial taxa that could assist the seaweed obtaining nutrients (e.g., nitrogen fixing bacteria) under elevated pCO_2_ conditions. These predictions, for both seaweed and gut microbiome, are limited to 16S taxonomic assignments and literature description and inherent reservations to this method will be considered.

## Methodology

### Experimental set-up

The experiment was performed at Centro de Ciencias do Mar (CCMAR) field station (Ramalhete) during the spring of 2014. Ambient (380 ppm CO_2_, and pH 8.16—global levels of today’s CO_2_ conditions) and elevated pCO_2_ (1,000) ppm CO_2_, and pH 7.86—the year 2100 predictions by IPCC, A1FI scenario (Houghton et al., 2001) conditions were controlled by two separate CO2 sensors systems. For acidified conditions, CO_2_ was injected in seawater deposits that provided seawater for the experimental units. In both systems salinity was 36, alkalinity 2,550 µmol kg^−1^ and seawater temperature 15 °C. Experimental units consisted of 3 L flowthrough mesocosms receiving each 30 L of seawater per hour. Experimental units were placed in one square meter tanks with 15 cm of the overflown seawater of the experimental units to stabilize temperature conditions in the units. *Sargassum muticum* and *Syniosoma nadejda* were sampled independently and kept isolated. Thus, the grazers were ‘naive’ and not previously on a diet containing *S. muticum*. After sampling, seaweeds and isopods were acclimated to ambient conditions in separate tanks for 1 week. A wet weight biomass of 1 g seaweed per experimental unit was used as a preliminary test and showed it did not affect the pH conditions in experimental units at the used volume and flow.

**Figure 1:**
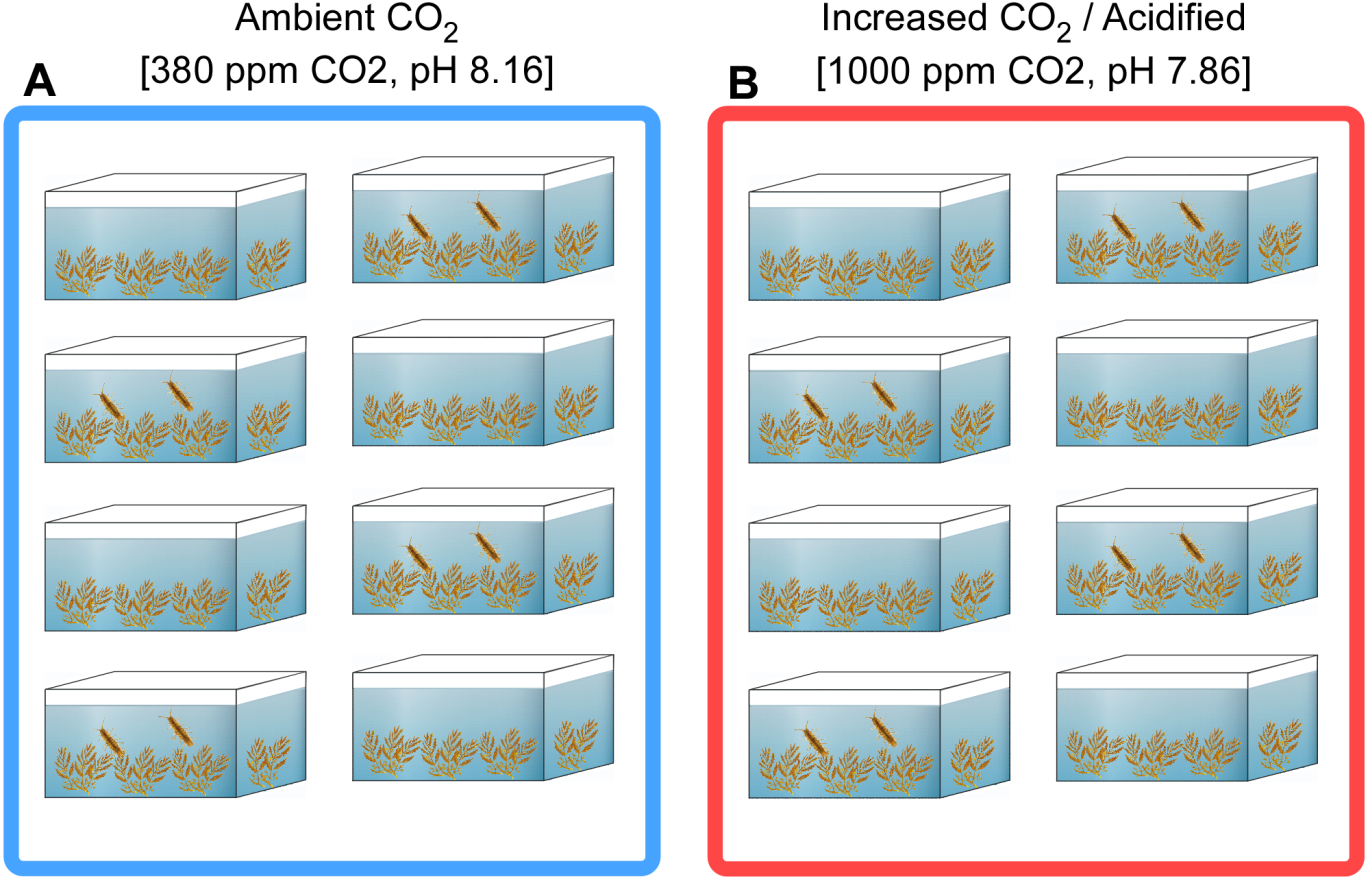
Schematic representation of the mesocosms experiment. (A) Ambient (380 ppm) and (B) acidified (1,000 ppm) conditions each with four 3 L experimental units only containing 1 g wet weight (WW) *S. muticum* and four 3 L experimental units containing 1 g WW *S. muticum* along with the grazer *S. nadejda* were placed randomly in each CO_2_ treatment. Each unit represented a replicate from which sample(s) (seaweed or seaweed and grazer) were taken.

The two factors considered for the experiment were (1) CO_2_ conditions (two levels: 380 and 1,000 ppm), and (2) host conditions (three levels: seaweed with grazers, seaweed without grazers, the grazers that fed on the seaweed). These were combined in a factorial design (all possible combinations of all levels of the two factors). The number of replicates within treatment combination was four. Therefore, in total, 24 samples (2 CO_2_ × 3 hosts × 4 replicates) were collected (Fig. 1). The experiment ran for three weeks which was estimated sufficient for microbiomes to reflect the applied conditions considering the high turnover rate of food in the grazer gut and the multiplication rate of bacteria. During this period the units were cleaned, twice a week, to avoid epiphyte overgrowth on the experimental unit walls. In each experimental unit, seawater pH and the calibration of the automated CO_2_ injection system was manually checked daily to make sure the pH was stable. At the end of the three weeks, the isopods and seaweeds were flash frozen, transported to the laboratory in liquid nitrogen and stored there at −80 °C until further processing.

### Hight-throughput sequencing of the microbiome

For both seaweeds and isopodes’s guts (also refered to as grazers, hereafter), DNA was extracted from all the 24 replicates using the Quick–gDNA kit (Zymo Research™, Irvine, CA, USA) according to the manufacturer protocol for “Solid Tissue Samples” (page 4 of the manual). Before extraction, isopods where dissected by removal of both ends and pulling out the intestinal tract. The total 16S rRNA was amplified using the universal primers 27F and 1492r with the following changes to the original protocol (Lane, 1991): an initial denaturation at 95 °C for 2 min, 35 cycles of denaturation at 95 °C for 20 s, annealing at 55 °C for 20 s, and extension at 72 °C for 90 s, with a final extension was at 72 °C for 3 min. The 25 µl reaction mixture contained 250 µM dNTPs, 0.6 µM of each primer, 1 × 2 PCR buffer mix, 2 µl of template DNA (with a final concentration of about 10 ng µl–1) and 0.3 µl of Taq polymerase (Advantage R2; Clontech, Mountain View, CA, USA). PCR products were cleaned using ExoFastAP enzyme following the Thermo Scientific™ protocol. Amplified DNA was sent to Molecular Research (MR DNA), Shallowater, Texas, where a nested–PCR was performed prior to sequencing. The modified 8 bp key–tagged primer 799F along with the reverse primer 1193R, covering the regions V5–V7 from 16S rRNA and amplifying a fragment of ∼400 bp, were used to avoid chloroplast cross amplification (Bodenhausen, Horton & Bergelson, 2013). PCR conditions were as follow: 95 °C for 3 min, 10 cycles of 95 °C for 20 s, 50 °C for 30 s, 72 °C for 30 s, and a final elongation of 72 °C for 3 min. All amplifications and sample preparation procedures were the same for both seaweeds and isopodes. Samples were pooled together in equal proportions based on their molecular weight (calculated based on the size of the amplicon) and DNA concentrations (using Qubit™; Invitrogen®, Carlsbad, CA, USA) and purified using calibrated Agencourt® AMPure® XP beads. DNA libraries were prepared by following Illumina TruSeq DNA library preparation protocol and paired–end (2 × 250 bp) sequencing performed at MR DNA (http://www.mrdnalab.com; Shallowater, TX, USA) on a MiSeq following the manufacturer’s guidelines.

Detailled protocols for sampling procedures, DNA extraction and PCR amplification mentioning all the important measures to avoid contamination, can be found in (Aires et  al., 2018).

### Sequence analysis and bioinformatics

A total of 3,204,094 partial 16S rRNA gene sequences were obtained from the 24 samples (i.e., two CO_2_ conditions X four replicates for seaweed in the presence and the absence of grazer and four grazer gut samples with two CO_2_ conditions). The bacterial community analyses were performed using Quantitative Insights into Microbial Ecology (QIIME version 1.8.0) software (Caporaso et al., 2010). Sequences were screened and filtered for a minimum read length of 350 bp (after reads were paired) and less than two undetermined nucleotides. Selected high-quality sequences were clustered into Operational Taxonomic Units (OTUs) within reads using denovo OTU picking method. Representative sequences for each OTU were selected using the “most-abundant” method and OTU sequence alignment was carried out using PyNAST (Caporaso et al., 2010) and Greengenes v.13.8 (McDonald et al., 2012). Taxonomic assignments were done using the UCLUST (Edgar, 2010) method with a 97% confidence threshold. To assign each OTU to the closest matching described taxon, searches were performed against the Greengenes taxonomy database v.13.8 for16S rRNA (McDonald et al., 2012), and sequences were putatively assigned to a described taxon with a minimum threshold of 0.001 (default value). Eukaryotes (i.e., chloroplasts and mitochondria) matching sequences were excluded from the OTU table in downstream analyses as well as rare OTUs (singletons and doubletons) and unassigned sequences (those sequences that did not match any of those from the Greengenes database, with a minimum threshold of 0.001).

Quality filtering resulted in 2,877,493 high-quality sequences, with an average of 119,896 ± 46,294 reads per sample, which were clustered into 42,730 unique operational taxonomic units (OTUs). The OTU table was rarefied to the minimum number of sequences (66,831). As a result, a total of 41,139 unique OTUs remained. Public access to the data can be done through: https://doi.org/10.6084/m9.figshare.5346316.v3.

All the statistical and diversity (alpha and beta) analysis were done using the filtered rarefied (to the minimum number of sequences—66,831) OTU table and considered significant at *P* < 0.05.

Alpha diversity indexes, including Chao *I* richness (Chao, 1984), observed number of species (OTUs) and Shannon diversity, were calculated using QIIME software. Bacterial community structure (beta diversity) was assessed by permutational multivariate analyses of variance (PERMANOVA) using Bray–Curtis dissimilarity matrices from square-root transformed data. PERMANOVA tested for differences among samples with different levels of *a priori factors*: Type of sample: Seaweed vs Grazer gut; for both CO_2_ treatments: Ambient vs Acidified; for Seaweed: Grazed vs Non-grazed, and the interactions among these factors. The homogeneity of multivariate dispersions (based on mean distance to group centroid for all groups within each factor) was tested using a resemblance based permutation test (PERMDISP). To visualize differences and to assess dissimilarity between samples, Canonical Analysis of Principal coordinates (CAP) plots were constructed to test the assignment/clustering of treatments interaction (*S.muticum* XGrazingxAcidification and Grazer gutxAcidification) as *a priori* factor. Similarities and dissimilarities in bacterial communities between acidification treatments were explored using, similarity percentage analyses (SIMPER). For those bacterial taxonomic groups that displayed a high contribution (concerning their differential abundances in the treatments being compared) for the differences between the grazer gut and the seaweed and the two CO_2_ levels, two-way analyses of variance (ANOVA) were performed (with the preliminary tests for normality and homogeneity of variances being implemented). Species (*S. muticum* and isopod) and acidification (CO_2_ ambient and elevated) were tested as factors affecting the structure/composition bacterial communities. For bacterial OTUs for which significant interaction were detected, a *post-hoc t*-test was implemented using a Bonferroni correction and a conservative alpha (considering the comparisons made: CO_2_ effect in *S. muticum*, CO_2_ effect on isopod gut), effect of type of tissue (seaweed/gut) in ambient CO_2_ and effect of type of tissue in elevated CO_2_ (*P* (*T* ≤ *t*) two tail < 0.0125).

All bacterial community structure statistical analyses were performed using the software program PRIMER-E + PERMANOVA v.6 (Clarke & Warwick, 1994; Clarke & Gorley, 2006).

## Results

### Bacterial communities associated with *S. muticum* and *S. nadejda’s* gut

Overall, 563 bacterial OTUs from 74 classes (apart from bacteria classified as ‘Other and non-ID’) distributed across 28 phyla (plus ‘Other and non-ID’) were identified. Among them, 551 bacterial OTUs distributed across 27 phyla (plus Other and non-ID) were associated with *S. muticum* (over all samples and treatments), compared to 450 OTUs distributed across 22 phyla (plus Other and non-ID) for gut biome. Note that those sequences labelled as “others” were ambiguous assignments by the classifier and “no-ID” sequences result from a good match with a reference sequence but that reference sequence is poorly defined (not named at a certain taxonomic level and below). Public access to the data can be done through: https://doi.org/10.6084/m9.figshare.5346316.v3.

Alpha diversity of associated bacterial communities (Table S1) was similar or slightly higher in *S. muticum* than in the grazer gut for Shannon index (2-way ANOVA, *F* = 2.507, *P* = 0.139), OTU richness (2-way ANOVA, *F* = 3.361, *P* = 0.092) and Chao 1 (2-way ANOVA, *F* = 6.226, *P* = 0.028), respectively. Acidification did not affect bacterial diversity at OTU level, for both seaweeds and isopodes gut, as estimated by diversity indexes: Shannon index (2-way ANOVA, *F* = 0.048, *P* = 0.831), unique OTU richness (2-way ANOVA, *F* = 0.178, *P* = 0.681) and Chao 1 (2-way ANOVA, *F* = 0.003, *P* = 0.960).

**Figure 2:**
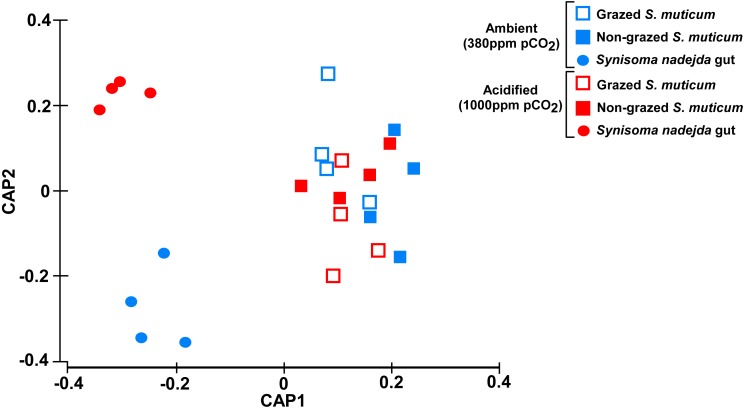
Community structure. Plot of canonical analysis of principal coordinates (CAP) based on Bray–Curtis distances calculated on square-root transformed bacterial abundances, showing the axes that best discriminate the bacterial assemblages across CO_2_ levels (blue-ambient *versus* red-acidified), grazing by *S. nadejda* on *S. muticum* (open squares-grazed seaweed vs filled squares-non-grazed seaweed) and the gut of the isopod on a diet of *S. muticum* (circles).

Despite the overall PERMANOVA results showing that bacterial community composition was significantly different for the different type of samples (grazed and non-grazed *S. muticum* and grazer gut, *P* = 0.001, Fig. 2, Table S2), predation (grazing) did not significantly change bacterial community structure of *S. muticum* (*P* = 0.372, Table S3A). The main differences among *S. muticum* (grazed and non-grazed) and the isopode gut (Table S2) were due to a higher abundance of *Bacteroidetes* (73.7%) associated to *S. muticum* (contributing 15.11% to the dissimilarity; SIMPER analysis) and to more *Proteobacteria* (47.2%) and *Planctomycetes* (32.4%) in the gut of *S. nadejda,* contributing 11.76 and 14.98% to the dissimilarity, respectively (SIMPER analysis).

Bacterial phyla-specific to host (refered in PERMANOVA analysis as “Type of sample”) and specific conditions are presented in Table S4.

**Figure 3:**
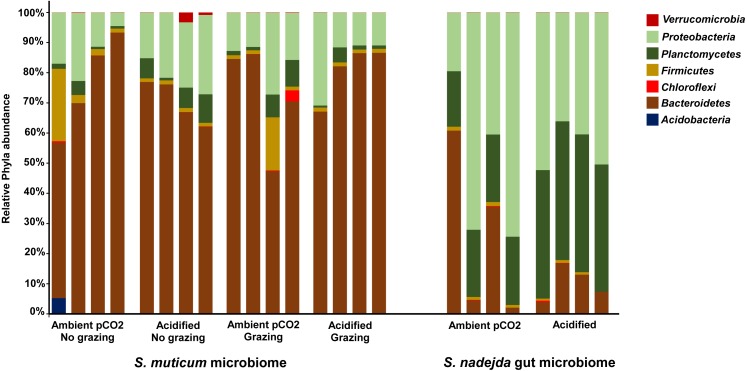
Host and treatment effects on associated bacteria phyla. Relative abundance and distribution of the bacteria phyla associated to the brown seaweed *Sargassum muticum,* without (No grazing) and with (grazing) *Synisoma nadejda* isopods, and the gut of the isopod after three weeks on a *Sargassum muticum* diet, under ambient (380 ppm) and elevated/acidified (1,000 ppm) CO_2_ conditions.

**Figure 4:**
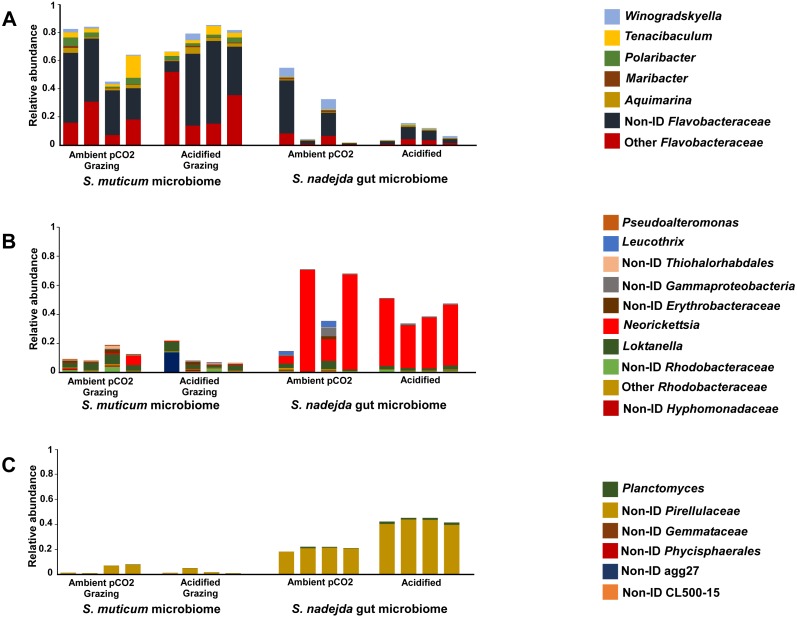
Relative abundance of the genera belonging to the main bacterial phyla. (A) Bacteroidetes, (B) Proteobacteria, and (C) Planctomycetes, associated with the brown seaweed *Sargassum muticum* grazed by *Synisoma nadejda* isopods (left side), and the gut microbiome of the isopod on a *Sargassum muticum* diet (right side), after three weeks under ambient (380 ppm; −CO_2_) and elevated/acidified (1,000 ppm; +CO_2_) CO_2_ conditions.

As mentioned before, grazing did not show significant effects on *S. muticum* bacterial community structure so, most of further analyses focused on the comparison of grazed *S. muticum* vs grazer gut. Grazed *S. muticum* associated bacterial communities were dominated by *Bacteroidetes* (75.4%, Fig. 3; *Flavobacteriia* 75.2%) and *Proteobacteria* (16.2%, Fig. 3; *Alphaproteobacteria* 12.2%), while the isopod gut communities were dominated by *Proteobacteria* (47.2%, Fig. 3; *Alphaproteobacteria* 43.5%), *Planctomycetes* (32.4%, Fig. 3; *Planctomycetia* 32.1%), and *Bacteroidetes* (17.7%, Fig. 3; *Flavobacteriia* 16.8%). *Flavobacteriales* (73.3%) was the most common bacterial order associated with *S. muticum*, while *Rickettsiales* (38.4%), *Pirellulales* (30.9%) and *Flavobacteriales* (16.8%) were the most abundant grazer gut-associated orders. The distribution of the bacterial genera belonging to the main phyla found for the two different systems and described above (*S. muticum*—*Bacteroidetes* and *S. nadejda* gut—*Proteobacteria* and *Planctomycetes*) showed a clear higher relative abundance of genera assigned as Non-ID and “other” *Flavobacteraceae* (Fig. 4A) for the seaweed microbiome and a isopode gut dominated by *Neorickettsialles* (Fig. 4B) and non-identified *Pirellulaceae* (Fig. 4C).

**Figure 5:**
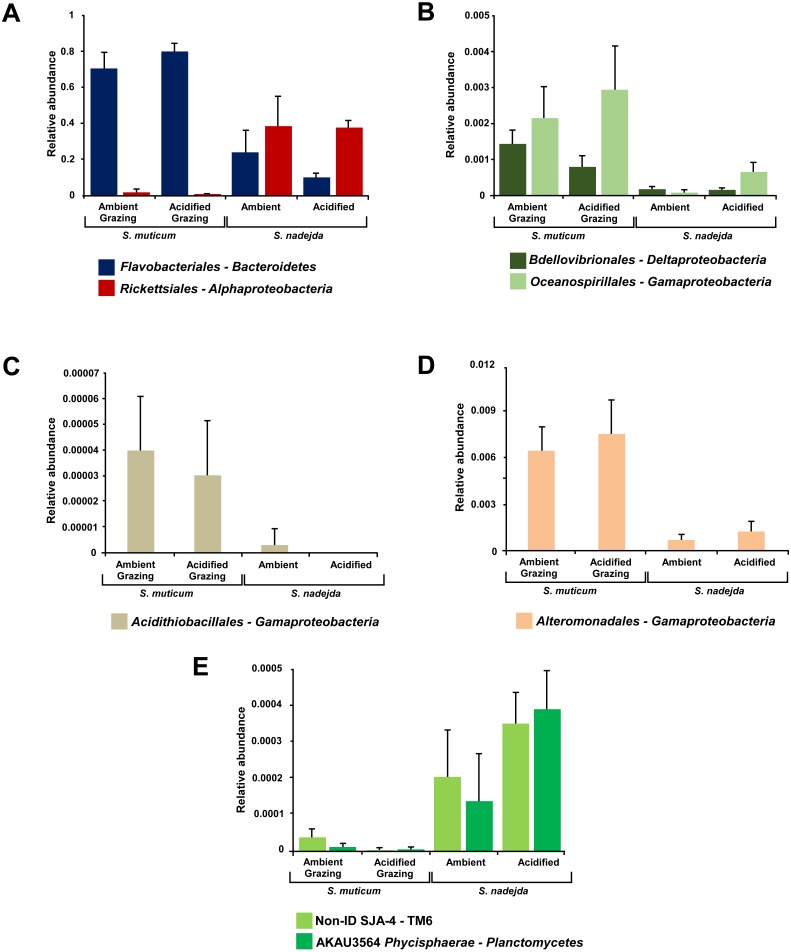
Mean relative abundances of bacterial classes, and respective orders (A, *Flavobacteriales* and Ricketsiales; B, *Bdellovibrionales* and *Oceanospirillales*; C, *Acidithiobacillales*; D, *Alteromonadales*; E, Non-ID *SJA-4* and *AKAU3564 Phycisphaerae*), significantly more abundant in either grazed *Sargassum muticum* or the gut of *Synisoma nadejda.* After three weeks under ambient (380 ppm) and elevated/acidified (1,000 ppm) CO_2_ conditions. Alpha = 0.05, error bars show standard error per treatment (*n* = 4).

Several bacterial orders were significantly more abundant within grazed *S. muticum* than in the isopod gut: *Flavobacteriales* from the phylum *Bacteroidetes* (2-way ANOVA, *F* = 49.124, *P* < 0.001); *Bdellovibrionales* from the class *Deltaproteobacteria* (2-way ANOVA, *F* = 13.221, *P* = 0.003); as well as *Acidithiobacillales* (2-way ANOVA, *F* = 22.589, *P* = 0.0005), *Alteromonadales* (2-way ANOVA, *F* = 15.285, *P* = 0.002), and *Oceanospirillales* (2-way ANOVA, *F* = 6.407, *P* = 0.026) from the class *Gammaproteobacteria* (Fig. 5)*.* In contrast, the isopod gut microbiome had higher abundances of *Rickettsiales* from the class *Alphaproteobacteria* (2-way ANOVA, *F* = 18.574, *P* = 0.001); the low abundance bacteria from the phylum *TM6*—class *SJA-4* (2-way ANOVA, *F* = 12.491, *P* = 0.004); and the low abundance order *AKAU3564*-*Phycisphaerae* from the phylum *Planctomycetes* (2-way ANOVA, *F* = 7.325, *P* = 0.019) than *S. muticum* in the presence of grazer (Fig. 5).

### Bacterial diversity and composition under acidification conditions

Acidification did not affect the overall bacterial composition (PERMANOVA, *P* = 0.093, Table S2) and in particular that associated with *S. muticum* (*P* = 0.056, *P* = 0.584, Table  S3B), but significantly affected the bacterial composition associated with the gut of *S. nadejda* (PERMANOVA, *p* = 0.022, Table S3B) (Fig. 2). However, under acidified conditions (and in the presence of grazers), the number of phyla in *S. muticum* bacterial community dropped from 26 to 17 (including one unidentified), compared to the ambient conditions (One-way ANOVA, *F* = 7.228, *P* = 0.009). In contrast, the number of phyla in the grazer gut-associated bacterial community under acidification treatment increased from 18 to 23 (including one unidentified), compared to the ambient conditions (One-way ANOVA, *F* = 1.923, *P* = 0.171).

The phyla specific to the microbiomes of *S. muticum* and gut of *S. nadejda,* under ambient *vs* acidified conditions, are presented in Table S4.

**Figure 6:**
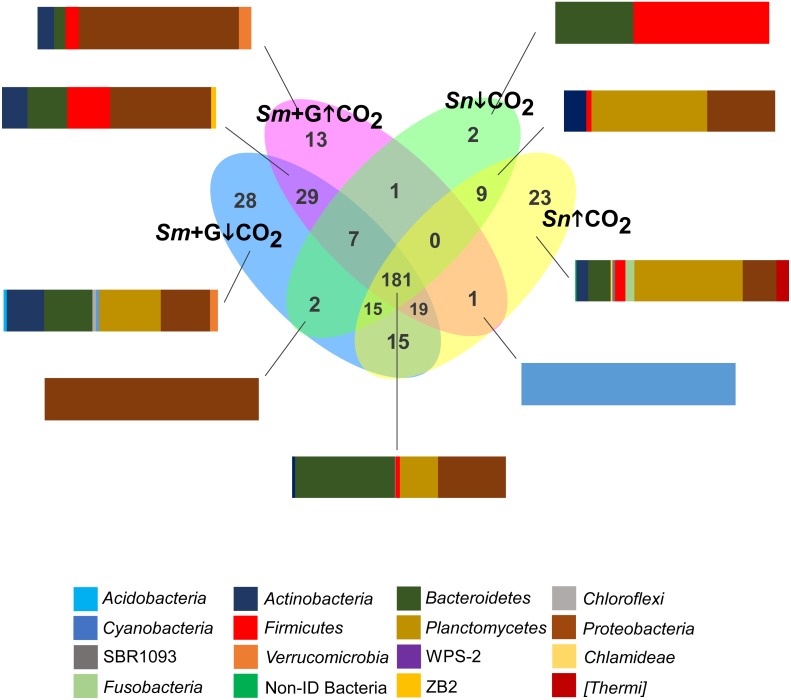
Core communities. Venn diagram representing the number of bacterial genera (present in at least 75% of samples) shared between the different CO_2_ treatments (ambient CO_2−_ ↓ CO_2;_ elevated/acidified CO_2−_ ↑ CO_2_) and associated to grazed *Sargassum muticum* (Sm + G), and the gut microbiome of the isopod *Synisoma nadejda* on a *Sargassum muticum* diet. The bar plots show the distribution of Phyla of selected intersections.

Some bacterial groups were unique to by their hosts under specific conditions (Fig. 6). The seaweed, in the presence of grazer and under the ambient conditions, had the highest number of unique bacterial OTUs (*n* = 28, 5%), while the grazer gut in the ambient conditions had the lowest number of unique bacterial OTUs (*n* = 2) (Fig. 6). *S. muticum* with the grazer present under the ambient conditions contained many OTUs belonging to *Planctomycetes* (3 OTUs, 24.8%), *Proteobacteria* (9 OTUs, 19.8%) and *Bacteroidetes* (9 OTUs, 19.5%) (Fig. 6). *S. muticum* with the grazer, but under acidification treatment, contained 13 unique OTUs, most of which belonged to *Proteobacteria* (7 OTUs, 74.9%) (Fig. 6). *Sphingobacterium* (*Bacteroidetes*) and *Caldicoprobacter* (*Firmicutes*) were unique to the gut of *S. nadejda* in the ambient conditions, compared to 23 OTUs unique to the grazer gut in the acidified conditions, a significant part of which belonged to *Planctomycetes* (2 OTUs, 50.7%) and *Proteobacteria* (8 OTUs, 15.8%) (Fig. 6).

A core bacterial community (present in at least 75% of samples) was composed of 181 bacterial OTUs (32.1%; including unidentified) (Fig. 6). Within this core bacterial community, the highest number of OTUs belonged to *Bacteroidetes* (*n* = 29; 55.3%), *Proteobacteria* (*n* = 90; 26.7%) and *Planctomycetes* (*n* = 7; 13.2%) (Fig. 5). Shared bacterial communities within *S. muticum* in the presence of grazer between the ambient and acidified conditions (*n* = 29; 5.2%) had the highest number of bacterial OTUs belonging to *Proteobacteria* (*n* = 14; 47.2%), *Firmicutes* (*n* = 8, 19.9%), and *Bacteroidetes* (*n* = 5; 18.5%) (Fig. 6). Shared bacterial communities in the gut of *S. nadejda* between the ambient and acidified conditions (*n* = 9; 1.6%), belonged mostly to *Planctomycetes* (*n* = 3; 54.8%) and *Proteobacteria* (*n* = 4, 32.3%) (Fig. 6).

**Figure 7:**
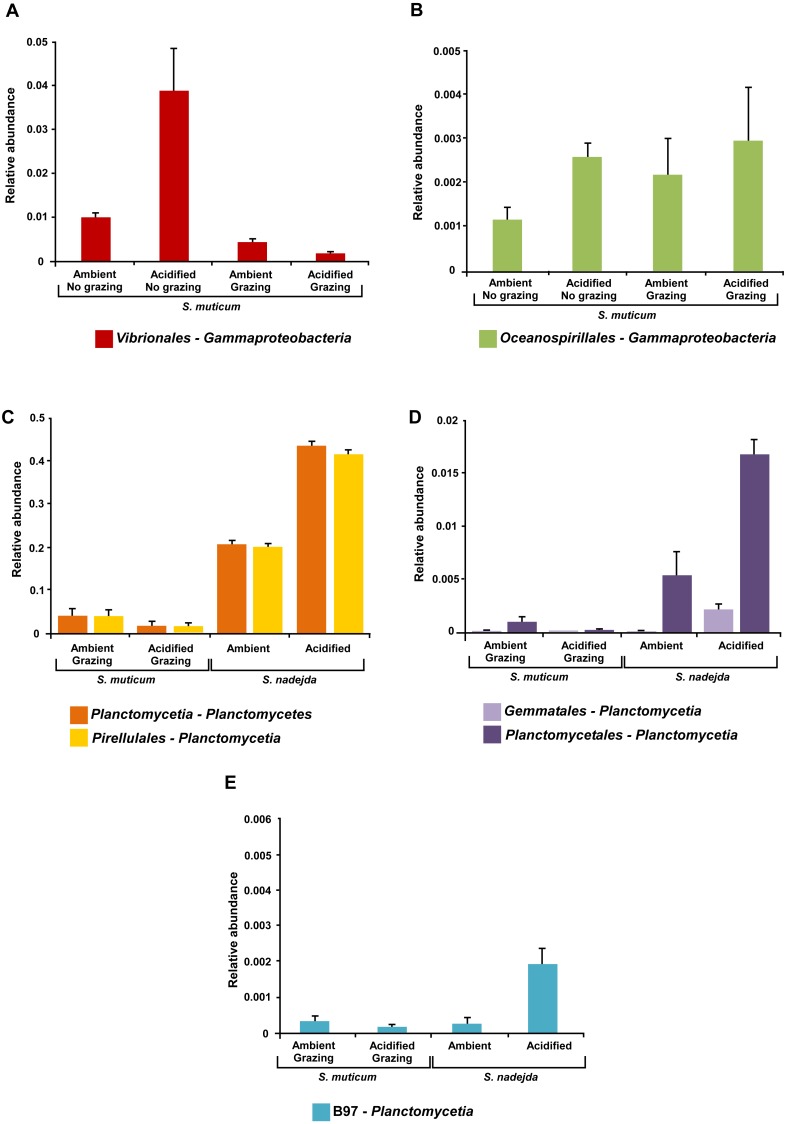
Mean relative abundances of associated bacterial orders. (A and B) *Sargassum muticum* under grazing/non grazing influence after three weeks under ambient (380 ppm) and elevated/acidified (1,000 ppm) CO_2_ conditions and (C–E) grazed *Sargassum muticum* and the gut of *Synisoma nadejda* after three weeks under ambient (380 ppm) and elevated/acidified (1,000 ppm) CO_2_ conditions, that responded to acidification, but for which a significant interaction between acidification and type of sample (seaweed or grazer gut) was observed. Alpha = 0.05, error bars show standard error per treatment (*n* = 4).

Under elevated CO_2_ treatment, relative abundances of *Gammaproteobacteria* from the orders *Oceanospirillales* and *Vibrionales* (particularly *Pseudoalteromonas*), increased on *S. muticum* but only significantly in the absence of grazers (One-way ANOVA, *P* = 0.037 and *P* = 0.047, respectively; Figs. 7A and 7B). Under acidification, the abundance of *Planctomycetia* associated with the grazer gut was significantly higher than at the ambient CO_2_ levels (Two-tailed *t*-test, *P* < 0.001), as was their abundance (at elevated CO_2_) in the grazer gut than on *S. muticum* (Two-tailed *t*-test, *P* < 0.001) (Fig. 7C). Deeper analyses of bacterial abundance within *Planctomycetia* revealed that all bacterial orders detected within this class responded to acidification treatment (Figs. 7C–7E). A significant effect was confirmed within the grazer gut under different CO_2_ conditions and between the seaweed and the grazer gut under increased CO_2_. At elevated CO_2_ levels, the abundance of *Pirellulales* (Two-tailed *t*-test, *P* < 0.001, Fig. 7C), *Planctomycetales* (Two-tailed *t*-test, *P* = 0.008, Fig. 7D) and *Gemmatales* (Two-tailed *t*-test, *P* = 0.009, Fig. 7D), were increased in the isopode gut. Alongside, under the acidification treatment, the abundance of the bacterial orders *Pirellulales* (Two-tailed *t*-test, *P* < 0.001, Fig. 7C), *Planctomycetales* (Two-tailed *t*-test, *P* < 0.001, Fig. 7D) and *Gemmatales* (Two-tailed *t*-test, *P* = 0.008, Fig. 7D), was significantly higher in the grazer gut than associated with the seaweed (in the presence of grazers). The class *C6-Planctomycetes* also exhibited a significant increase in abundance under elevated CO_2_ conditions, but only within the grazer gut (Two-tailed *t*-test, *P* = 0.039) (Fig. 6). This was mostly due to the significant increase of bacteria from the order *d113* (two-tailed *t* test, *P* = 0.039) (Fig. 6). The abundance of *d113* under acidified conditions was significantly higher in the grazer gut than within *S. muticum* (two-tailed *t* test, *P* = 0.018) (Fig. 6). Overall, under acidification treatment, *Planctomycetes* increased in abundance (particularly within *Planctomycetia*) in the grazer gut from 20.9% to 43.8% (1-way ANOVA, *F* = 306.663, *P* < 0.001).

## Discussion

The results presented in this experimental study demonstrated that acidification affected specific bacterial groups, but hardly influenced the overall microbiome of the invasive brown seaweed *S. muticum*. In contrast, acidification caused significant changes in the gut microbiome of a native isopod consumer, *S. nadejda*. Interestingly, acidification increased abundances of *Planctomycetia* in the gut of *S. nadejda* and of *Oceanospirillales* and *Vibrionales* associated to *S. muticum*, raising hypotheses about their functional role under these conditions.

The guts of isopods are populated by symbiotic bacteria (e.g., Zimmer & Bartholmé, 2003; Wang, Brune & Zimmer, 2007; Fraune & Zimmer, 2008; Eberl, 2010) that assist in food utilization by the host (Zimmer, 2002; Zimmer et al., 2002; Zimmer & Bartholmé, 2003; Fraune & Zimmer, 2008). Because the diversity of gut bacterial communities is shaped by host diet (Tietjen, 2014), changes in seaweed characteristics and its microbiome, as a result of ocean acidification, were expected to affect the gut microbiome of the grazer with possible fitness consequences. Here, we documented that the most striking change resulting from acidification was a significantly increased abundance of *Planctomycetes* in the gut of *S. nadejda*, more specifically due to an increase of Non-ID *Pirellulaceae.* In this case, as seaweed microbiome was not overall affected by OA, these general changes in the grazer’s gut cannot be directly attributed to changes in its food microbiome. Although the functions of *Pirellulaceae* are hardly known, its presence was documented in the gastrointestinal tract of fish (Parris et al., 2016) and as part of the resident microbiomes of marine copepods but in very low abundance in starved specimens (Moisander, Sexton & Daley, 2015). *Planctomycetes* constituted the second most prevalent phylum (after *Proteobacteria*) in the gut of *S. nadejda*. These bacteria are known to widely colonize aquatic and terrestrial ecosystems (Lage & Bondoso, 2014) and were, until recently, considered environmental organisms. However, *Planctomycetes* have been reported associated to the gut of, not only marine taxa (Singh & Reddy, 2014), but also terrestrial herbivores (Fuerst & Sagulenko, 2011), mammals (Frey et al., 2006) and even humans (Cayrou et al., 2013).

In this study, acidification strongly increased the abundance of *Planctomycetes* in the bacterial gut communities of *S. nadejda* compared to ambient conditions. An increase of this Phylum in response to simulated OA has also been shown in sandy sediments (Currie et al., 2017). Although it is clear that in our study this was a consequence of acidification, the lack of seawater samples does not allow us to determine whether the observed *Planctomycetes* increase was due to acidification of the seawater or a direct response of the grazer’s gut to acidification. This phylum was also abundant (the third most abundant) in the bacterial community associated to *S. muticum*. These widespread bacteria have been often found associated to macroalgae (Lage & Bondoso, 2014), among which *Caulerpa taxifolia* (Meusnier et al., 2001) and the kelp *Laminaria hyperborea* (Bengtsson, Sjøtun & Øvreås, 2010). Bacteria from the phylum *Planctomycetes* are suggested to have potential benefits for their hosts, through their ability to mineralize organic molecules into inorganic compounds that match the nutritional requirements of macroalgae (Lage & Bondoso, 2014). *Planctomycetes* are also proposed to function as “slow-acting decomposers of organic matter” (i.e., algal polymer degradation; Bodelier & Dedysh, 2013) and important contributors to the global nitrogen cycle (i.e., anammox *Planctomycetes*, Fuerst & Sagulenko, 2011). So, the presence of these bacteria in the isopod gut might also be related to the ingestion of *S. muticum.* Its increased abundance (particularly those from the order *Pirellulales*) could also be a response to the change of the seaweed nutritional value under acidification, which likely increased its photosynthetic rate driven by high CO_2_ and consequent C/N ratio shifts (Cornwall, Revill & Hurd, 2015; Briggs, 2017). An increase in *Planctomycetes* abundance could provide the isopod with an elevated algal digestion capacity to compensate the highly carbonated food. Again, with the lack of control samples as starving isopods (empty guts) we can only raise new hypotheses about the direct (through its microbiome) or indirect (through its nutritional value/quality) influence of the food in the grazer’s gut microbiome. Yet, there is no doubt that acidification resulted in a significant overall change in the isopod gut microbiome that is not directly related to bacterial community shifts of *S. muticum*, which did not occur for the seaweed at elevated CO_2_.

Another important change resulting from acidification was a significant increase in *Oceanospirillales* (Non-ID *Oceanospirillaceae*) and *Vibrionales* (*Pseudoalteromonas*) associated with *S. muticum* in the absence of grazers*.* They all belong to *Gammaproteobacteria* which have been previously found in association with various seaweed species (e.g., Patel et al., 2003; Huggett et al., 2006). Members of the class *Gammaproteobacteria* are known to produce biologically active metabolites that mediate antifungal (Barbieri et al., 2001), antifouling (i.e., *Alteromonas*, *Pseudomonas*; Maki et al., 1988; Holmstrom, Rittschof & Kjelleberg, 1992; Avelin Mary et al., 1993; Holmström & Kjelleberg, 1999) and antibacterial activities (Hentschel et al., 2001). There are no studies documenting the effect of acidification on these bacteria in seaweeds, but environmental samples showed a high increase of *Gammaproteobacteria* under acidification, in particular from the order *Oceanospirillales* (Currie et al., 2017)*.* Bacteria from the order *Oceanospirillales* are heterotrophic and capable of degrading complex organic compounds (Garrity et al., 2005; Goffredi, Johnson & Vrijenhoek, 2007). These bacteria use organic matter as food source and their increase in *S. muticum* under acidification might be opportunistic and related to the carbon content increase in the seaweed, and consequent seaweed enrichment, due to elevated CO_2_ levels.

Increased abundance of *Vibrionales* has often been associated with stressed and diseased marine invertebrates and they are also known as coral pathogens (Bourne & Munn, 2005; Bourne et al., 2008; Sunagawa et al., 2009; Meron et al., 2011). *Vibrionales* were also responsible for a number of infections in humans and animals (Vezzulli et al., 2016), and identified as potential pathogens of sablefish larvae (Schulze et al., 2006) and bivalve mollusks (Asplund et al., 2014). Interestingly, it has been shown that under low pH, the coral-associated pathogen *Vibrio sp.* increased in abundance (Meron et al., 2011), while the blue mussel pathogen *Vibrio tubiashii* became more infectious (Asplund et al., 2014). In this study, among the *Vibrionales* that experienced a significant increase were predominantly *Pseudoalteromonas.* While certain members of the genus *Pseudoalteromonas* were reported to have antibacterial activity in corals, providing it with defense against potential pathogens (Shnit-Orland, Sivan & Kushmaro, 2012), multiple studies identify *Pseudoalteromonas* as opportunistic pathogens of marine organisms (Liu et al., 2010; Song et al., 2012; Wang et al., 2012). Bacteria affiliated to the genus *Pseudoalteromonas* were found associated to the kelp *Laminaria japonica* affected by two different diseases (holle-rotten disease and red spot disease) (Sawabe et al., 1998; Gachon et al., 2010) where its isolation and posterior reinfection resulted in observable symptoms (Wang et al., 2008). Nevertheless, particular bacteria that may be otherwise commensal, under stress of the seaweed host, can become saprophytic (Egan et al., 2013) and this could hypothetically be the case of the *Pseudoalteromonas* found in *S. muticum* under acidification. Ocean acidification could potentially result in shifts from healthy associated bacterial communities within seaweeds towards a higher prevalence of pathogenic bacteria and/or increased vulnerability to disease. Unfortunately, our taxonomic assignments and our amplicon sequencing approach does not provide more detailed insights into possible bacterial pathogenicity.

*Flavobacteriales* from the *Bacteroidetes* phylum were among the bacterial groups that were significantly more abundant in association with *S. muticum* than in the isopod gut. *Bacteroidetes* colonize marine and freshwater environments widely (Thomas et al., 2011), populating a wide variety of surfaces, including macroalgae (Beleneva & Zhukova, 2006; Staufenberger et al., 2008) and marine sediments (Devine, Pelletreau & Rumpho, 2012). *Bacteroidetes* were isolated from *Caulerpa taxifolia* (Meusnier et al., 2001), *Ulva australis* and the red alga *Delisea pulchra* (Longford et al., 2007), suggesting that these typical marine bacteria are common seaweed associates (Tujula et al., 2010). This phylum represents some of the most abundant marine bacteria (Glöckner et al., 1999; Simon, Glöckner & Amann, 1999; Cottrell & Kirchman, 2000) and plays an important role as degraders of complex organic matter (Church, 2008). *Flavobacteriia*, the most prevalent class detected within the seaweed microbiome, are known to produce enzymes for polymer degradation (Fenchel, 2012). Most *Flavobacteriia* are able to degrade cellulose, chitin, proteins, and nucleic acids (Kirchman, 2002; Fenchel, 2012). However, *Bacteroidetes* are also related to stress conditions and often found in diseased corals (Barneah et al., 2007), as in the case of *Porites compressa* which when exposed to low pH showed an increase of disease-associated *Flavobacteriia* (Thurber et al., 2009). *Flavobacteriales*, the most widely represented *Flavobacteriia* order within *S. muticum*, are mostly associated with degradation of complex particle biomacromolecules, as well as algal debris (Kirchman, 2002), more specifically proteins, agars, xylan, fucoidan, cellulose, and chitin (Devine, Pelletreau & Rumpho, 2012). Bacteria from the genera *Aquimarina* and *Tenacibaculum* were among the most frequent OTUs and occur free-living in marine environments (Nedashkovskaya et al., 2006) and fixed to the surfaces of marine organisms (Suzuki et al., 2001). Bacteria from the genus *Tenacibaculum* are thought to induce morphogenesis in algae and possibly enhanced seaweed growth (Matsuo et al., 2003; Matsuo et al., 2005).

In this study, acidification resulted in a small (non-significant) decrease of seaweed-associated *Flavobacteriales* and just in the absence of grazer. This contrasts with a study conducted on biofilms from the Great Barrier Reef, which reported that with decreasing pH there was an increase in the relative abundance of *Flavobacteriales* (*Flavobacteriaceae*) (Witt et al., 2011). However, Witt et al. (2011) focused on natural biofilms on glass slides whereas biofilms from living organisms are the result of environment and host communication and may be differently influenced by any fluctuations that might have occurred in the seawater bacterial communities. Nevertheless, the lack of seawater bacterial community analysis in our study may limit the interpretation of some of these results. The decrease of these common seaweed associates (Hollants et al., 2013; Egan et al., 2013), although non significant, may result from the instablity of environmental pH changes. Natural seaweed bacterial assemblages can be disrupted by environmental pressures (Marzinelli et al., 2015) and that could contribute to the initial response of *S. muticum* to acidification.

Seaweed consuming iguanas, which have adapted to use macroalgae as their primary resource, were found to host a large propotion of *Bacteroidetes* in their gut when compared to terrestrial related species (Hong et al., 2011). Seaweed polysaccharides, many with sulfated sugars that are absent in terrestrial plants, are easily hydrolized by *Bacteroides* spp. present in the gastrointestinal tract (Shah & Gharbia, 1993). In particular, these bacteria have been described as contributing to the degradation of brown algal polysaccharides in the gastrointestinal tract of limpets (particularly *Flavobacteriia*; Dudek et al., 2014) and other gastropods that are seaweed consumers (Cardoso et al., 2012). As discussed above, *Bacteroidetes* were among the most abundant bacteria associated with *S. muticum* and, as shown on copepods (Moisander, Sexton & Daley, 2015), these were the third most abundant bacterial phylum in the intestinal tract of *S. nadejda. Bacteroidetes* are within the most abundant phyla in three different copepod species, for both starving and full gut specimens (Moisander, Sexton & Daley, 2015). These authors also found these bacteria to be the most abundant in seawater samples which lead to the assumption that they were not “gut permanent residents” but instead colonizers from the seawater. In our case, the lack of seawater samples and starved isopodes (empty gut) limit the interpretation of our results, so its relatively high abundance in *S. nadejda* gut might be both related with seawater and/or food.

In contrast to *Bacteroidetes* and *Gammaproteobacteria* prevailing within *S. muticum*, *Alphaproteobacteria* (*Rickettsiales*), as well as bacteria from the phylum *TM6* (the class *SJA-4*) were more abundant in the grazer gut microbiome for both ambient and acidified conditions. *Alphaproteobacteria* were also reported as one of the most prevalent Classes in marine copepods (Moisander, Sexton & Daley, 2015). Bacteria from the order *Rickettsiales*, which dominated the gut of *S. nadejda,* are known as pathogenic to humans and animals (Perlman, Hunter & Zchori-Fein, 2006), and were found in the intestinal tract of infected isopod *Armadillidium vulgare* (Dittmer et al., 2016) and several other isopode species (Wang, Brune & Zimmer, 2007). *Neorickettsia* is known as a parasite to which the invertebrates usually serve as vectors (Greiman et al., 2014). This aspect is worth further investigation once the possibility of these herbivores to function as pathogen vectors may have consequences on human health if they are able to pass them along to some edible algae where they feed on.

The experimentally controlled mesocosm data allowed us to isolate variables to infer their effects, even though they are not able to fully reconstruct the dynamic conditions of nature, as documented for corals that had different bacterial communities in laboratory and field (Kooperman et al., 2007; Meron et al., 2011). So, the cause of the observed bacterial community shifts can be easily identified but may not mimick exactly the processes in the field. Another limitation to realistic predictions is that global acidification may be happening quicker than IPCC models predicted. So, pH and CO_2_ conditions used in this and several other studies may not be realistic and underestimate the effects of OA that can be more catastrophic than what is expected to be in the next 100 years (the maximum IPCC prediction) (Thurber et al., 2009; Meron et al., 2011). However, those changes would happen over a large period of time and not over the course of three weeks, as in our experiment.

The results of this study demonstrate that bacterial communities associated within an isopod—seaweed predator–prey system are dynamic and responsive to changes in acidification. The observed changes in the associated bacterial communities of the seaweed and the grazer gut might be a type of acclimation that facilitates tolerance and survival, as suggested by Meron et al. (2011). The capacity of organisms to accommodate and modify a highly diverse microbiota may influence the fitness and success of the host and enhance its ability to survive changing environmental conditions (Morrow et al., 2015). Further research is required to better understand the processes and conditions under which different associated bacteria can increase tolerance of the host organisms to various disturbances.

## Conclusion

The responses of bacteria associated with *S. muticum* and the gut of *S. nadejda* revealed in this study suggest that worst case acidification scenarios may not greatly affect overall bacterial community composition and diversity, but might affect specific bacterial groups.

Contrarily to what was expected, acidification to expected levels seems to have less significant impact on seaweed bacterial ecology than other environmental stressors, such as increased temperature (Mensch et al., 2016). However, specific groups had significant abundance shifts in seaweeds under acidification as was the case for *Oceanospirillales* and *Vibrionales* (mainly *Pseudomonadales*). The former might be related to the possible increase of the C/N ratio in the seaweed under acidified conditions and the latter, commonly found associated with diseased seaweeds, could be an indicator that acidification enables an increase of opportunistic/pathogenic bacteria. Unexpectedly, no particular bacteria increased abundance that could assist the seaweed in obtaining nutrients (like nitrogen fixing bacteria) under a higher carbon availability regime.

In contrast with the seaweed host, high CO_2_ levels globally changed the bacterial community associated to the isopod gut, particularly the abundance of members of the Class *Planctomycetia*. However, as no significant changes occurred in the global seaweed microbiome, the overall shift in the grazer gut bacterial community cannot be directly attributed to bacterial changes in the food. Instead, we can hypothesize that the food “quality” changed at elevated CO_2_ triggering a shift in the isopod gut *Planctomycetia* allowing it to better digest the seaweed and compensate the shifted C/N ratio as seaweed becomes less nutritional under acidification, as previously hypothesized. The generality of these findings must be addressed in further studies with other species.

*Bacteroidetes* (mainly *Flavobacteriia*), previously isolated and commonly found associated with other seaweed species (Huggett et al., 2006; Burke et al., 2011a; Lachnit et al., 2011), were the most dominant phyla, suggesting that they account for core roles in the metabolism of *S. muticum*. Both in ambient and acidified conditions, the isopod gut bacterial community was dominated by *Neorickettsia (Alphaproteobacteria)*, for which invertebrates are usually the vectors. The pathogenicity of these bacteria has already been shown in other invertebrate species (Greiman et al., 2014) and the potential of isopod species as possible vectors for these bacteria has already been suggested (Yuksel, Thompson & Adams, 2006) and should be further investigated.

Concluding, our results show that after only three weeks of simulated acidification, bacterial communities associated to a seaweed host, when ungrazed, and to an isopod grazer gut, show shifts in composition. These bacterial community changes were particular and specific in the seaweed and only occurred when ungrazed, but were large and global community shifts in the isopod grazer gut. We hypothesized that these may have potential consequences for seaweed health and isopod food digestion. The observed changes in the gut microbiome of the grazer seem to be a reflection of changes in the seaweed chemistry rather than its microbial composition.

##  Supplemental Information

10.7717/peerj.4377/supp-1 Supplemental Information 1 Supplemental Tables

## Additional Information and Declarations

### Data Availability

The following information was supplied regarding data availability: Engelen, Aschwin (2018): Acidification + Grazing_Effects_Microbiome. figshare. https://doi.org/10.6084/m9.figshare.5346316.v3.
