# Supplementary material for: Acidification increases abundances of Vibrionales and Planctomycetia associated to a seaweed-grazer system: potential consequences for disease and prey digestion efficiency
Source: PeerJ. 2018 Mar 30;6:e4377. doi: 10.7717/peerj.4377 (PMC5880178; doi:10.7717/peerj.4377)
Supplement: Supplemental Information 1 [file peerj-06-4377-s001.docx]

**Supplementary Data**

**Table S1.** Alpha diversity indexes (Chao1, Observed OTUs and Shannon diversity index) of bacterial communities of each replicate of *S. muticum* and *S. nadejda* gut under the influence of the different treatments

| **Sample ID** | **Chao1** | **Observed OTUs** | **Shannon Div Index** |
| --- | --- | --- | --- |
| **Sm1-G- CO_2_** | 13777.862 | 6538 | 7.694 |
| **Sm2-G- CO_2_** | 16321.957 | 7977 | 8.426 |
| **Sm3-G- CO_2_** | 16588.578 | 8181 | 8.196 |
| **Sm4-G- CO_2_** | 13015.667 | 6614 | 7.225 |
| **Sm1-G+ CO_2_** | 13450.298 | 5823 | 6.207 |
| **Sm2-G+ CO_2_** | 12626.508 | 7015 | 8.139 |
| **Sm3-G+ CO_2_** | 20779.573 | 9169 | 8.490 |
| **Sm4-G+ CO_2_** | 18184.070 | 7552 | 7.897 |
| **Sm1+G- CO_2_** | 15920.414 | 7873 | 8.444 |
| **Sm2+G- CO_2_** | 13085.994 | 6267 | 6.911 |
| **Sm3+G- CO_2_** | 20701.812 | 10821 | 9.591 |
| **Sm4+G- CO_2_** | 14950.894 | 6432 | 6.931 |
| **Sm1+G+ CO_2_** | 8460.372 | 3780 | 5.113 |
| **Sm2+G+ CO_2_** | 17697.615 | 9435 | 9.091 |
| **Sm3+G+ CO_2_** | 12796.553 | 5977 | 6.629 |
| **Sm4+G+ CO_2_** | 15063.619 | 7038 | 7.146 |
| **GG1- CO_2_** | 11046.235 | 5793 | 7.175 |
| **GG2- CO_2_** | 7898.099 | 4177 | 3.847 |
| **GG3- CO_2_** | 12371.221 | 7172 | 8.287 |
| **GG4- CO_2_** | 7372.279 | 3773 | 4.422 |
| **GG1+ CO_2_** | 9181.274 | 4222 | 5.624 |
| **GG2+ CO_2_** | 15105.188 | 7185 | 7.563 |
| **GG3+ CO_2_** | 13835.134 | 6427 | 6.930 |
| **GG4+CO_2_** | 10558.879 | 5066 | 6.152 |

**Sm**- *S. muticum*, **-CO_2_** (Ambient, 380 ppm), **+CO_2_** (Acidified, 1000 ppm), **GG**- Grazer (*S. nadejda*) gut, G- (no grazer present), G+ (grazer present),

# Table S2. Results of PERMANOVA main test based on square-root transformed rarefied OTU table and Bray-Curtis distances.

| **Source of Variation** | **Pseudo-F** | **P (perm)** |
| --- | --- | --- |
| Type of sample | 4.1431 | **0.001** |
| Acidification | 1.2753 | 0.093 |
| Type of sample x Acidification | 1.3654 | **0.010** |

**H0**: there are no differences in the distribution of OTUs in different species and acidified conditions. H0 rejected if: **p** < **0.05**. Bold face values are significant at P < 0.05. P-values based on 999 permutations. Type of sample refers to *S. muticum* grazed tissue. *S. muticum* non-grazed tissue and grazer gut (independently of CO_2_ conditions).

**Table S3. A)** Pairwise PERMANOVA comparisons for bacterial communities associated to *S. muticum* (*Sm*) grazed and non-grazed and grazer gut within the term “Type of sample”, **B)** Pairwise PERMANOVA comparisons for the interaction “Type of sample” and “Acidification”.

**A.**

| **Term: Grazing** | **P(perm)** |
| --- | --- |
| Non-Grazed *Sm* vs Grazed *Sm* | 0.372 |
| Grazed *Sm* vs Grazer gut | **0.001** |
| Non-Grazed *Sm* vs Grazer gut | **0.001** |

**B.**

| **Term: Grazing x Acidification** | |
| --- | --- |
| ***Factor: Grazing*** | |
| *Level: Non-Grazed Sm* | |
| Ambient vs Acidified | **P(perm)** |
|  | 0.056 |
| *Level: Grazed Sm* | |
| Ambient vs Acidified | 0.584 |
| *Level: Grazer gut* | |
| Ambient vs Acidified | **0.022** |
| ***Factor: Acidification*** | |
| *Level: Non-Acidified* | |
| Non-Grazed *Sm* vs Grazed *Sm* | 0.168 |
| Non-Grazed *Sm* vs Grazer gut | **0.033** |
| Grazed *Sm* vs Grazer gut | **0.04** |
| *Level: Acidified* | |
| Non-Grazed *Sm* vs Grazed *Sm* | 0.232 |
| Non-Grazed *Sm* vs Grazer gut | **0.027** |
| Grazed *Sm* vs Grazer gut | **0.036** |

**H0**: there are no differences in the distribution of OTUs in different species and locations. H0 rejected if: **p** < **0.05**. Bold face values are significant at P < 0.05. P-values based on 999 permutations.

**Table S4.** Bacterial phyla specific to *Sargassum* *muticum*. the gut of *Synisoma* *nadejda* grazer and/or the grazer and acidification treatment.

| **Phylum** | ***S. muticum*** | | | | ***S. nadejda* gut** | |
| --- | --- | --- | --- | --- | --- | --- |
|  | **No grazer** | | **With grazer** | |  |  |
|  | **CO2-** | **CO2+** | **CO2-** | **CO2+** | **CO2-** | **CO2+** |
| *Gemmatimonadetes* | + | + | + | + | - | + |
| *Nitrospirae* | + | + | + | + | - | + |
| *Fusobacteria* | + | + | + | - | + | + |
| *Tenericutes* | + | + | + | **-** | - | + |
| *BRC1* | + | + | + | **-** | - | - |
| *Chlamydiae* | - | + | + | - | + | + |
| *OP3* | + | - | + | - | - | + |
| Non-classified *BH180-139* | + | + | - | - | - | - |
| *Caldithrix* | + | - | + | - | - | - |
| *Fibrobacteres* | + | - | + | - | - | - |
| Non-classified *NKB19* | + | - | - | - | - | - |
| *SAR406* | + | - | - | - | - | - |
| *GNO2* | - | - | - | - | - | + |
| *WS3* | - | - | + | - | - | + |
